# Effect of a Phenotype-Tailored Lifestyle Intervention on Caloric Intake and Macronutrient Composition in Adults with Obesity: A Secondary Analysis of the PHENO-Diet Trial

**DOI:** 10.3390/nu18193159

**Published:** 2026-09-25

**Authors:** Maria Antonia Espinosa, Lindsey Sefried, Jose Villamarin, Lizeth Cifuentes, Thomas Fredrick, Diego Anazco, Sima Fansa, Jamie Friend, Thomas M. Rieck, Michael Camilleri, Maria D. Hurtado Andrade, Matthew M. Clark, Donald D. Hensrud, Andres Acosta

**Affiliations:** 1 Precision Medicine for Obesity Program, Division of Gastroenterology and Hepatology, Department of Medicine, Mayo Clinic, Rochester, MN 55905, USA; espinosamonteros.maria@mayo.edu (M.A.E.); sefried.lindsey@mayo.edu (L.S.); hurtado.mariadaniela@mayo.edu (M.D.H.A.); hensrud.donald@mayo.edu (D.D.H.); 2 Healthy Living Program, Integrative Medicine and Health, Mayo Clinic, Rochester, MN 55905, USA; 3 Clinical Enteric Neuroscience Translational and Epidemiological Research, Division of Gastroenterology and Hepatology, Department of Medicine, Mayo Clinic, Rochester, MN 55905, USA; 4 Division of Endocrinology, Diabetes, Metabolism, and Nutrition, Department of Medicine, Mayo Clinic, Jacksonville, FL 32224, USA; 5 Department of Psychiatry and Psychology, Mayo Clinic, Rochester, MN 55905, USA; 6 Division of Endocrinology, Diabetes, Metabolism, and Nutrition, Department of Medicine, Mayo Clinic, Rochester, MN 55905, USA; 7 Division of General Internal Medicine, Department of Medicine, Mayo Clinic, Rochester, MN 55905, USA

**Keywords:** macronutrients, precision medicine, lifestyle intervention, phenotypes, obesity, dietary intake

## Abstract

Background/Objectives: A 12-week trial demonstrated that phenotype-tailored lifestyle intervention (PLI) was associated with greater weight loss than standard lifestyle intervention (SLI); however, differences in dietary patterns between groups were not examined. Methods: This secondary analysis evaluated caloric and macronutrient intake at 12 weeks in adults with obesity (BMI ≥ 30 kg/m^2^), classified into four energy-balance phenotypes (abnormal satiation, reduced postprandial satiety, low energy expenditure, emotional eating) (*n* = 77) who underwent PLI or SLI. Dietary intake was assessed by 24-h recalls and verified by a blinded registered dietitian. Results: Groups were compared at 12 weeks adjusting for baseline values. PLI was associated with lower total energy intake than SLI (1273 ± 182 vs. 1894 ± 176 kcal/day; *p* = 0.018), due to lower carbohydrate (464 ± 76 vs. 818 ± 73 kcal/day; *p* = 0.002) and fat intake (479 ± 76 vs. 697 ± 73 kcal/day; *p* = 0.036); absolute protein intake did not differ. However, protein comprised a greater proportion of energy intake in PLI (26.8 ± 1.6%) than SLI (19.8 ± 1.5%; *p* = 0.003), and dairy protein contributed more to total energy in PLI (6.8 ± 0.8% vs. 3.7 ± 0.7%; *p* = 0.004). Conclusions: PLI was associated with lower energy intake, mainly due to reduced carbohydrate and fat intake, and a higher relative contribution of protein, consistent with phenotype-based recommendations rather than macronutrient prescriptions. These findings suggest a potential role for personalized dietary approaches in obesity management. However, the non-randomized design does not allow causal inferences.

## 1. Introduction

Obesity is a complex and multifactorial disease with a rising global prevalence [1,2]. Projections suggest that by 2030, nearly 1 in 2 adults will be living with obesity in the United States [3]. Lifestyle interventions, which per standard-of-care encompass an energy deficit of ~500–750 kcal/day and ≥150 min/week of moderate-intensity activity, are central to achieving and maintaining weight loss [4]. Yet, standard-of-care lifestyle modification (SLI) achieves inconsistent long-term success in most individuals, mainly because metabolic and behavioral adaptations to weight loss make it difficult to sustain engagement over time [5]. Furthermore, weight loss outcomes in response to SLI vary significantly across individuals [6]. The goal of a phenotype-tailored approach is to address this variability by aligning treatment with each individual’s physiological and behavioral drivers of weight gain.

Phenotype-tailored lifestyle interventions (PLI) seek to account for interindividual differences in the behavioral and physiological traits (e.g., emotional eating, abnormal satiation, reduced postprandial satiety, low energy expenditure) by adapting dietary and physical activity prescriptions to an individual’s measurable behavioral and physiological traits [7,8]. This targeted approach has the potential to produce greater and more durable weight loss than the traditional “one-size-fits-all” lifestyle model.

In a clinical trial comparing 3-month weight loss outcomes between PLI and SLI, PLI was associated with 1.7-fold greater weight loss. This superior weight loss was accompanied by a fivefold larger reduction in total energy intake in PLI versus SLI, as assessed by 24-h dietary recall [7]. PLI recommendations were tailored to specific behavioral and physiological traits or phenotypes and emphasized food-based behavioral and dietary adjustments rather than prescriptive macronutrient targets. Consequently, changes in the relative contributions of carbohydrate, fat, and protein to total energy intake, both between treatment arms and across phenotypes, have not been reported. Understanding these differences is clinically relevant, as shifts in macronutrient distribution represent a possible mechanism for the benefits observed with phenotype-tailored approaches and could inform the development of more precise dietary prescriptions for obesity management.

This analysis is based on the parent PHENO-Diet trial, which showed that PLI resulted in greater weight loss than SLI [7]. Weight loss outcomes are not re-evaluated in this analysis; instead, the study characterizes macronutrient composition and dairy protein contribution, which were not published in the parent trial. The dietary strategies used in PLI, including high protein, volumetric, and time-restricted strategies, are each well-established in the nutrition literature. The novelty of this analysis is the characterization of the macronutrient changes that are associated with applying these strategies by phenotype, rather than identifying new nutritional mechanisms.

To address this gap, we conducted a secondary analysis comparing energy intake and macronutrient composition between PLI and SLI at 12 weeks, adjusting for baseline values, and explored how these differences varied across obesity phenotype subgroups. Total caloric intake was a pre-specified secondary endpoint of the parent trial; the macronutrient-composition and dairy-protein analyses reported here were not pre-specified and are therefore exploratory and hypothesis-generating. We hypothesized that PLI would produce greater reductions in total energy intake compared to SLI, accompanied by distinct shifts in macronutrient composition reflecting adherence to phenotype-specific dietary guidance.

## 2. Materials and Methods

### 2.1. Study Design and Participants

This study is a secondary analysis of the PHENO-Diet trial, a 12-week, non-randomized intervention conducted at the Mayo Clinic in Rochester, Minnesota. This trial used a non-randomized, two-phase design: a single multidisciplinary team delivered the standard lifestyle intervention (SLI) in Phase 1 (August 2020–February 2021) and the phenotype-tailored intervention (PLI) in Phase 2 (March 2021–February 2022). The phases were separated in time to limit the influence of the single intervention team’s increasing familiarity with the phenotype-tailored protocol on the standard arm, consistent with the proof-of-concept, pilot-feasibility design of the parent study [7]. Adults aged 18 to 65 years with a body mass index (BMI) >30 kg/m^2^ were enrolled. The study was approved by the Institutional Review Board (IRB # 19-007485) at Mayo Clinic, Rochester, MN, and was registered at ClinicalTrials.gov (NCT04073394). All participants provided written informed consent prior to study participation. Total daily caloric intake was a pre-specified secondary endpoint of the parent trial [7]; macronutrient composition, relative macronutrient distribution and dairy protein contribution are new analyses reported here for the first time.

Participants underwent validated obesity phenotype testing [9] at baseline and 12 weeks after intervention. After an overnight fast, phenotype classification was determined with a comprehensive assessment that included body composition analysis by dual-energy X-ray absorptiometry scans (Hologic QDR 4500A, Bedford, MA, USA), resting energy expenditure (REE) measurement by indirect calorimetry (ParvoMedics, Sandy, UT, USA), solid gastric emptying assessed after a standardized 1339 kJ (320 kcal) radiolabeled breakfast followed by scintigraphy (SPECT/CT), an ad libitum meal, and multiple behavioral and appetite questionnaires throughout the day. The standardized radiolabeled breakfast included two eggs, one slice of brown bread and 120 mL of skim milk (320 kcal, 32% protein, 35% fat, 33% carbohydrate). The ad libitum meal consisted of vegetable lasagna (326 g box: 420 kcal, 17 g protein, 38 g carbohydrate, and 22 g fat); vanilla pudding (99 g carton: 130 kcal, 1 g protein, 21 g carbohydrate, and 4.5 g fat); and skim milk (236 mL carton: 90 kcal, 8 g protein, 13 g carbohydrate, and 0 g fat) [7]. Fasting glucose, HbA1c, insulin, LDL- and HDL-cholesterol, triglycerides, and high-sensitivity C-reactive protein were measured in the Mayo Clinic clinical laboratory using standard, clinically validated assays. Blood pressure and heart rate were recorded using standard clinical techniques.

Four distinct phenotypes were classified based on previously established cut-offs reflecting key aspects of energy balance regulation [10]: abnormal satiation, defined as calories to maximal fullness during an ad libitum meal greater than 3741 kJ (894 kcal) for females and greater than 5757 kJ (1376 kcal) for males; reduced postprandial satiety, defined as gastric emptying half-emptying time less than 101 min for females and less than 86 min for males; low energy expenditure, defined as a ratio of measured to predicted resting energy expenditure less than 0.96 in females and less than 0.94 in males; and emotional eating, defined as Hospital Anxiety and Depression Scale for anxiety (HADS-A) score of 7 or greater for both males and females, the cutoff used to identify this phenotype in the validated framework [9,10]. Obesity phenotypes were not mutually exclusive, and participants could meet criteria for more than one. In the PLI arm, participants meeting more than one phenotype received dietary recommendations aligned to their dominant phenotype.

### 2.2. Standard Lifestyle Intervention

SLI consisted of a structured two-day program followed by 12 weeks of support from a multidisciplinary team of obesity experts. Recommendations included a daily energy deficit of 500 kcal per day based on measured resting energy expenditure at baseline, 150 min of moderate physical activity per week, and a goal of 10,000 daily steps [11]. Participants also received 12 individual sessions with a health and wellness coach.

### 2.3. Phenotype-Tailored Lifestyle Intervention

PLI recommendations were designed to target physiological or behavioral mechanisms contributing to excess weight in each individual. A set of interventions was derived for each specific phenotype based on prior nutritional research [10]. The phenotype-specific recommendations and their physiological rationale are summarized in Table S8. Diet, exercise, and behavioral components of the intervention were assigned independently based on the phenotype criteria a participant met, so a participant could receive components for more than one phenotype at the same time. A single choice was required only for diet, since the abnormal satiation and reduced postprandial satiety diets were mutually exclusive; in these cases, the phenotype for which the participant’s measured value fell at a more extreme percentile relative to the normative cutoffs [9] was assigned as dominant for diet selection. This determination was made at the baseline visit, before any dietary outcome data were available.

Participants with abnormal satiation were advised to adopt a low-calorie, volumetric diet enriched with insoluble fiber, fruits, and vegetables within an 8-h daytime time-restricted eating window, with healthy second servings of fruits or vegetables allowed. The goal was to suppress hunger signaling for longer periods, maximize gastric distension to induce fullness using high-volume low-energy-density foods, and channel the physiological need for second servings toward low-calorie options. Participants with reduced postprandial satiety were advised to follow a low-calorie high-protein diet with a pre-meal protein shake or healthy protein snack before each meal and to eat three to five structured meals per day. The purpose was to deliver a preload with protein to increase early release of gastrointestinal satiety hormones such as glucagon-like peptide-1 (GLP-1) and peptide YY (PYY) and to slow gastric emptying, targeting the accelerated gastric emptying that defines this phenotype. Participants with low energy expenditure were advised to follow a low-calorie high-protein diet, three meals per day, and a post-workout protein shake or snack, combined with a supervised exercise program of resistance training and high-intensity interval training (HIIT) four to five times per week with weekly physiotherapist check-in. The purpose was to increase lean mass and total energy expenditure, given that lean mass is the primary driver of REE. Participants with emotional eating were advised to follow a low-calorie diet of three structured meals per day with no snacks, to avoid using food as a coping mechanism. They participated in 12 weekly interactive video telehealth group sessions incorporating cognitive-behavioral therapy, emotion regulation, resiliency strategies, mindfulness, self-compassion, harm reduction and stimulus control, co-led by a clinical health psychologist and a health and wellness coach.

### 2.4. Dietary Intake Collection

Dietary intake was evaluated using three paper-based 24-h recalls. The first diary was distributed following comprehensive phenotyping and collected at the baseline visit marking the start of the intervention. A second diary was distributed at week 4 and returned at week 8; participants could complete it on any day within that interval. A third diary was completed at week 12.

Participants were educated on how to record all foods and beverages consumed during the previous 24 h. Dietary intake was self-reported using self-administered paper-based 24-h recalls. At the baseline visit, study staff provided standardized in-person instruction: recording all foods and beverages from the previous 24 h, estimating portion sizes with household measures, indicating homemade versus commercial preparation, and noting brand or restaurant sources. Completed diaries were returned to study staff and logged into Cronometer [12].

### 2.5. Energy and Macronutrient Analysis

Macronutrient composition and total energy intake (kcal/day) were analyzed with Cronometer™ (web version; Cronometer Inc., Revelstoke, BC, Canada), a dietary platform that integrates nutrient profiles from standardized sources such as United States Department of Agriculture (USDA) and the Nutrition Coordinating Center Database (NCCDB) [12]. Cronometer’s calorie and macronutrient outputs have been shown not to differ significantly from the Nutrition Data System for Research (NDSR), the gold standard in dietary assessment [13], and the platform has been used for dietary tracking and data collection in recent clinical studies [14,15,16].

Total caloric intake and macronutrients were analyzed as absolute values (kcal/day) and as percentages of total daily energy intake. A registered dietitian, blinded to group allocation, reviewed every diary for completeness and internal consistency, clarified incomplete or ambiguous entries using standard portion references, and entered all items into Cronometer. The dietitian’s review verified the completeness and internal consistency of each recall; intakes were not screened for biological implausibility, and no formal implausibility cutoff (for example, a Goldberg-type cutoff) was applied.

Given prior evidence that dairy intake may influence satiety and metabolic outcomes [17,18,19], protein intake was subcategorized by source to evaluate the potential contribution of dairy protein to study endpoints. Dairy protein was defined as protein derived from milk, yogurt, cheese and whey-based protein sources. Whey from protein shakes was included when explicitly specified by the participant. Plant-based milk alternatives such as soy or almond milk were not classified as dairy. Energy from dairy protein was calculated using a standard conversion of 4 kcal per gram of protein and was reported as a percentage of total energy intake.

### 2.6. Outcomes

The primary endpoint was the difference in total energy intake (kcal/day) and absolute macronutrient intake (carbohydrates, fat, and protein) in kcal/day between PLI and SLI at 12 weeks. Secondary endpoints included differences in macronutrient composition (percentage of total energy from carbohydrate, fat, and protein) between study groups. The relative contribution of dairy protein (kcal from dairy protein/total kcal) was also compared between groups.

Exploratory subgroup analyses compared energy intake and macronutrient composition at week 12 between PLI and SLI within each obesity phenotype subgroup (abnormal satiation, reduced postprandial satiety, emotional eating, low energy expenditure) adjusting for covariates (age, sex, baseline weight and baseline intake).

### 2.7. Statistical Analysis

All analyses were conducted using JMP Pro 18 (SAS Institute Inc., Cary, NC, USA). Statistical significance was set at a two-sided *p*-value less than 0.05.

Baseline continuous variables were assessed for normality using the Shapiro–Wilk test. Normally distributed variables were compared using Student’s *t*-test; non-normally distributed variables were compared using the Wilcoxon rank-sum test. Categorical variables were compared using chi-square or Fisher’s exact test as appropriate.

Total energy intake (kcal/day), absolute macronutrient intake (kcal/day), relative macronutrient composition (percent of total energy) and dairy protein energy (percent of total energy) were analyzed using linear mixed-effects models fitted by restricted maximum likelihood, using a compound symmetry covariance structure. Models included a random intercept for participant and fixed effects for intervention group (PLI vs. SLI), visit (baseline, mid-intervention, week 12), and a group-by-visit interaction, with age, sex, and baseline weight as covariates. Missing data were handled under a missing-at-random (MAR) assumption. Because MAR cannot be verified directly, we tested robustness with two sensitivity analyses: a complete-case analysis of covariance (ANCOVA) in week-12 completers, and a jump-to-reference analysis imputing missing PLI week-12 values at the observed SLI mean, a conservative departure from MAR.

From these models, least-squares means and standard errors were obtained for each group at every visit. Between-group differences were estimated as least-squares-means contrasts (PLI minus SLI) at each visit and are reported with 95% confidence intervals. Because several dietary outcomes were tested, the week 12 between-group *p*-values were adjusted for multiplicity using the Benjamini–Hochberg false discovery rate (FDR), applied separately within the primary outcome family (total energy, carbohydrate, fat, and protein intake) and the secondary outcome family (carbohydrate, fat, protein, and dairy-protein contribution to total energy). The phenotype-subgroup analyses were exploratory; for these, the Benjamini–Hochberg procedure was applied across all 16 subgroup-by-outcome comparisons. Differences at baseline and mid-intervention are reported descriptively with 95% confidence intervals.

Linear regression models were used within each obesity phenotype subgroup to compare week 12 energy and macronutrient intake between PLI and SLI, adjusting for baseline intake, age, sex, and baseline weight. Assumptions of linearity, normality of residuals, and homogeneity of variance were assessed using diagnostic plots. Because the phenotype subgroups were not mutually exclusive, these subgroup analyses were not independent and were considered exploratory.

Baseline characteristics are reported as means and standard deviations or medians and interquartile ranges where appropriate. All subsequent values are presented as covariate-adjusted least-squares means and standard errors (LSM ± SE).

## 3. Results

### 3.1. Study Participants

Between July 2020 and August 2021, 211 adults with obesity were screened and 165 were assigned based on the date of their study enrollment (non-randomized) to either phenotype-tailored lifestyle intervention (PLI) or standard lifestyle intervention (SLI). In this full cohort, most participants were women (81%) and White (92%), with a mean age of 43.9 years and a mean BMI of 38.3 kg/m^2^. Complete baseline characteristics of the full cohort have been previously reported [7].

For this secondary analysis, 77 participants who completed a 24-h dietary recall at baseline and had at least one additional recall during the intervention period were included (Figure 1), of whom 56 had a recall at mid-intervention (PLI: 22; SLI: 34) and 56 at week 12 (PLI: 27; SLI: 29). Baseline demographic and clinical characteristics were similar between groups. Baseline characteristics of the 77 included and 88 excluded participants were comparable (Table S6). The week-12 difference in energy intake was robust across sensitivity analyses: a complete-case ANCOVA (n = 56) gave an adjusted PLI–SLI difference of −631 kcal/day (95% CI −917 to −346; *p* < 0.001), and a jump-to-reference analysis imputing missing PLI week-12 values at the observed SLI mean gave −487 kcal/day (95% CI −755 to −218; *p* < 0.001), both consistent with the primary mixed-model estimate of approximately −621 kcal/day (Table S7). No significant differences were observed in resting energy expenditure, anxiety scores, or prevalence of obesity phenotypes (all *p* > 0.05; Table 1). Baseline total energy intake and macronutrient composition were similar between groups (Table 2).

### 3.2. Energy Intake and Macronutrient Composition

At 12 weeks, total energy intake was lower in PLI (1273 ± 182 kcal/day) compared to SLI (1894 ± 176 kcal/day; *p* = 0.018; Figure 2A; Table 3), based on least-squares means from the mixed-effects model; the adjusted between-group difference was Δ −621 kcal/day (95% CI −1086 to −156). Model-derived reductions from baseline were greater in PLI (−1394 ± 211 kcal) compared to SLI (−666 ± 200 kcal; *p* = 0.014).

Carbohydrate intake at 12 weeks was significantly lower in the PLI compared to the SLI (464 ± 76 vs. 818 ± 73 kcal/day; *p* = 0.002; Figure 2B), representing a between-group difference (Δ) of −354 kcal (95% CI −548 to −160). Model-derived reductions from baseline were −430 kcal in PLI and −84 kcal in SLI.

Fat intake was also lower in PLI than SLI (479 ± 76 vs. 697 ± 73 kcal/day; *p* = 0.036; Figure 2C; Δ −218 kcal/day, 95% CI −410 to −26). Protein intake did not differ between groups (321 ± 29 vs. 359 ± 28 kcal/day; *p* = 0.306; Figure 2D; Δ −38 kcal/day, 95% CI −110 to +35). However, protein represented a larger proportion of total energy in PLI versus SLI (26.8 ± 1.6% vs. 19.8 ± 1.5%; *p* = 0.003; Figure 3C; Δ +7.0%, 95% CI +3.1 to +10.9), corresponding to a model-derived increase from baseline of +6.9% in PLI compared with +0.8% in SLI. At 12 weeks, energy from dairy protein contributed a greater proportion of total energy in PLI than in SLI (6.8 ± 0.8% vs. 3.7 ± 0.7%; *p* = 0.004; Figure 3D; Δ +3.1%, 95% CI +1.1 to +5.1), corresponding to a model-derived increase of +5.2% in PLI and +1.9% in SLI. Carbohydrate intake also contributed a smaller proportion of total energy in PLI than SLI at 12 weeks (38.3 ± 2.3% vs. 44.4 ± 2.2%; Δ −6.1%, 95% CI −11.8 to −0.4; *p* = 0.047; Figure 3A).

### 3.3. Phenotype-Specific Intake Changes

In the analyzed cohort, 34 of 77 participants (44.2%) met criteria for more than one phenotype (SLI 33.3%, PLI 57.1%). At week 12, PLI was associated with significantly lower energy intake compared to SLI in three of four phenotype subgroups (Figure 4). The greatest reductions were observed in the low energy expenditure subgroup (1181 ± 155 vs. 2157 ± 176 kcal/day; Δ –976 kcal [95% CI −1418 to −534]; *p* = 0.002) and in the abnormal satiation subgroup (1179 ± 228 vs. 1990 ± 223 kcal/day; Δ –810 kcal [95% CI −1330 to −291]; *p* = 0.019). Participants with low energy expenditure were advised to follow a high-protein diet supplemented with post-workout protein and a structured resistance and high-intensity interval training program, targeting both caloric reduction and lean mass preservation. Participants with abnormal satiation were advised to follow a low-calorie volumetric diet enriched with fiber and vegetables within an 8-h time-restricted eating window, designed to reduce total energy intake by promoting earlier fullness.

The emotional eating subgroup also showed significantly lower adjusted energy intake in PLI compared to SLI (714 ± 133 vs. 1312 ± 120 kcal/day; Δ –598 kcal [95% CI −910 to −286]; *p* = 0.014). Participants with emotional eating were advised to follow a balanced low-calorie diet of three structured meals per day without snacks, combined with mindfulness, resilience training and cognitive-behavioral strategies.

In the reduced postprandial satiety subgroup, energy intake did not differ significantly between groups (1271 ± 254 vs. 1363 ± 342 kcal/day; Δ –91 kcal [95% CI −933 to +750]; *p* = 0.839; Table S5). Participants with reduced postprandial satiety were advised to follow a high-protein diet with pre-meal protein shakes and three to five structured meals per day, targeting meal timing and protein distribution rather than direct caloric restriction.

## 4. Discussion

In this secondary analysis of a non-randomized, proof-of-concept clinical trial, we examined how a phenotype-tailored lifestyle intervention (PLI) differed from a standard lifestyle intervention (SLI) in dietary composition at 12 weeks. PLI was associated with greater reductions in total energy intake than SLI, largely through lower carbohydrate intake, with a contribution from lower fat intake. In exploratory subgroup analyses, this pattern appeared in three of four phenotype subgroups (low energy expenditure, emotional eating, and abnormal satiation) but not in the reduced postprandial satiety subgroup. Consequently, the proportion of total energy from protein significantly increased by approximately 35% in PLI, with dairy protein accounting for part of this shift. As the energy intake values were obtained through self-reported dietary recalls, they reflect the reported energy intake, instead of measured energy intake; therefore, the extent of the reduction should be interpreted accordingly.

Obesity management requires dietary counseling that is personalized according to the patient’s individual needs, such as appetite, fullness and eating habits. The PLI incorporates this approach with a formal phenotyping process that identifies food-related behaviors and traits and provides recommendations without placing any emphasis on the amount of macronutrients in a patient’s diet. The SLI does not provide a generic diet either, as it prescribes a daily caloric deficit of 500 kcal based on REE measurement and includes regular wellness coaching and self-monitoring. Differences in macronutrient composition between groups may reflect the specific effect of phenotype-guided guidance beyond an already individualized program.

The macronutrient changes in PLI likely reflect the synergistic effect of phenotype-specific dietary, exercise, and behavioral components. Because these components were delivered simultaneously, the specific contribution of dietary individualization cannot be isolated. PLI did not prescribe specific macronutrient ratios, yet participants consistently reduced carbohydrate intake and increased the relative contribution of protein. When dietary advice is tailored to patients’ physiology and behavior, advice may be more readily understood, and adherence may improve. However, some phenotype-specific recommendations, particularly pre-meal protein shakes for the reduced postprandial satiety subgroup, may have influenced macronutrient composition within the PLI, and this should be kept in mind when interpreting the protein findings.

High-protein diets have been shown to enhance satiety and support fat loss while preserving lean mass, partly via increased postprandial secretion of GLP-1 and PYY [20,21]. However, these mechanisms remain hypotheses drawn from prior literature rather than findings of this analysis. An 8-week trial in 121 women with obesity comparing both low carbohydrate and higher protein diets found that only the lower-carbohydrate intervention arms improved postprandial satiety; higher protein alone had no effect [22]. That trial did not evaluate the timing and administration of protein, which may be critical factors. Furthermore, lower-carbohydrate diets can attenuate the drop in energy expenditure that typically follows weight loss [23], and high-quality low-carbohydrate dietary patterns emphasizing plant-based sources are associated with less long-term weight gain than patterns rich in refined carbohydrates and added sugars [24]. These effects may be particularly relevant in individuals with reduced postprandial satiety, in whom impaired postprandial satiety signaling is a defining feature of the phenotype [9]. At 12 weeks, participants in the PLI group derived approximately 6.9 percentage points more of their energy from protein, a relative increase of 35% from baseline. Prior studies comparing diets with approximately 20% versus 25 to 30% protein have reported greater fat loss and better lean mass preservation with higher protein intake [25], suggesting this magnitude of change may be clinically meaningful.

Caloric recommendations alone can miss the individual drivers of obesity. Phenotype-tailored lifestyle interventions address this heterogeneity by first identifying each participant according to their dominant obesity mechanism and then tailoring dietary and behavioral strategies accordingly [10]. Pre-meal protein supplementation may enhance GLP-1 and PYY release in individuals with reduced postprandial satiety [21,22] and reduce appetite [26]. In contrast, time-restricted volumetric, fiber-rich meals may reduce energy intake by promoting earlier fullness [27,28], making this approach suitable for patients with the abnormal satiation phenotype. In exploratory subgroup analyses, greater caloric reductions with PLI than SLI were observed in three of four phenotypes, consistent with the possibility that aligning dietary guidance to individuals’ underlying physiology can support adherence to caloric targets. The phenotype-specific findings should be interpreted with caution. Even though differences remained significant after correction for multiple comparisons (Benjamini–Hochberg), the subgroups were small, and as preliminary results from a proof-of-concept trial they cannot establish causality and warrant replication.

These findings have implications for how caloric targets are framed and delivered in clinical practice. Clinical guidelines for the management of obesity recommend a daily energy deficit of 500 to 1000 kcal based on estimated energy requirements [4]. However, uniform caloric targets overlook the different mechanisms that drive excess energy intake across individuals. In this study, the reported reduction in caloric intake with PLI exceeded common guideline-based caloric deficit targets in three of four phenotype subgroups; however, because dietary intake was self-reported, further research is needed to determine if energy deficits were achieved. Addressing the mechanism underlying excess energy intake in each patient, rather than restricting calories uniformly, may be key to achieving more meaningful dietary change. Phenotype-tailored dietary interventions may improve adherence to a caloric restriction by working with individual physiology and eating behaviors rather than applying uniform nutrient targets. In clinical practice, this approach offers a practical framework for personalizing obesity management.

By week 12, PLI participants also increased the proportion of total energy from dairy protein. This may reflect dietary shifts in response to phenotype-specific guidance that emphasized convenient, high-quality protein sources. A meta-analysis of 13 randomized clinical trials in 750 adults found that consuming more than 500 mL of dairy per day increased satiety and fullness while reducing hunger [18]. Dairy proteins such as whey and casein stimulates the release of satiety-promoting hormones through bioactive peptides [29], which may partly explain this pattern. This remains a hypothesis based on previous findings in the literature, but dairy protein was an exploratory rather than a pre-specified outcome, and this finding does not establish that dairy intake caused the observed benefits.

Energy intake did not decrease significantly in the reduced postprandial satiety subgroup, likely reflecting both limited statistical power and the nature of the phenotype-specific guidance. PLI recommendations for this subgroup focused on meal timing and protein distribution rather than direct caloric restriction, which may require more than 12 weeks to produce measurable reductions in total energy intake.

This study has several strengths. Obesity phenotypes were identified using a validated comprehensive assessment [9], and dietary data were verified by a registered dietitian blinded to group assignment, reducing the risk of measurement error. Dietary recalls were collected at three timepoints, allowing for longitudinal assessment of dietary change. Mixed-effects models adjusted for covariates strengthened comparability between groups despite the non-randomized design. Completion rates were high in both arms, limiting attrition bias.

Limitations include the non-randomized design and modest sample sizes, which reduce statistical power and limit generalizability. Only 47% of the parent cohort contributed dietary data, as dietary recall was a secondary endpoint and was not designed or powered as a primary dietary assessment. Included and excluded participants were broadly comparable, although the analyzed cohort contained a somewhat higher proportion of participants with abnormal satiation and reduced postprandial satiety; because the primary comparison concerns overall intake between PLI and SLI and the phenotype-specific analyses are exploratory, this modest imbalance is unlikely to affect the main findings. Missing follow-up recalls were handled under a missing-at-random assumption that could not be verified directly; sensitivity analyses that deliberately departed from this assumption yielded consistent results, although a missing-not-at-random mechanism cannot be excluded. The sequential two-phase design also means that the intervention team had completed delivery of the SLI before beginning PLI; provider familiarity with dietary counseling and phenotype-specific guidance may therefore have increased between phases, and this learning effect cannot be separated from the phenotype-specific intervention in the current design.

Emotional eating was defined by the HADS anxiety subscale (HADS-A ≥ 7), per the validated energy-balance framework [9,10]. Although HADS-A reflects anxiety, it correlates with the Three-Factor Eating Questionnaire (TFEQ-21) emotional-eating domain in adults with obesity [30].

Because the study design was non-randomized and recruited the two groups in sequential periods, the observed differences cannot be attributed solely to the intervention; unmeasured confounders between the recruitment periods may also have contributed. In addition, the intervention team provided SLI first; therefore, improved behavioral counseling experience may have also contributed to the observed differences independently of phenotype tailoring and cannot be isolated from it. The 12-week follow-up does not allow conclusions about longer-term sustainability of these dietary changes. Dietary intake was assessed by a single 24-h recall at each time point, which may not fully represent habitual intake and is subject to recall bias. Because intake varies substantially from day to day, a single recall may not capture habitual intake, increasing measurement error and the risk of misclassifying dietary change; this is particularly relevant because the primary outcomes are self-reported. Repeated recalls or objective intake biomarkers would reduce this error and are recommended for future studies. The low intakes reported in some subgroups are consistent with under-reporting, which is common with 24-h recalls in adults with obesity during weight-loss interventions; because no implausibility cutoff was applied and the phenotype subgroups were small, these subgroup estimates should be regarded as exploratory, and the primary comparison rests on the full analyzed cohort rather than any individual subgroup. Meal timing and eating patterns were not recorded. The phenotypes were not mutually exclusive, introducing potential overlap that complicates interpretation of phenotype-specific analyses. The study sample was predominantly White and of high socioeconomic status, and these findings may not apply to more diverse populations. Certain PLI recommendations, particularly the emphasis on protein for the reduced postprandial satiety phenotype, likely influenced macronutrient-specific findings. Moreover, because adherence data were not collected, it was not possible to assess the degree to which participants followed their phenotype-specific recommendations or link adherence to the observed dietary changes. Future studies should include dietary assessment as a primary endpoint with a structured completion process, use multiple recalls or objective biomarkers such as urinary nitrogen, and prospectively document reasons for non-completion to enable more rigorous missing data analysis.

Future studies should test phenotype-tailored dietary approaches in randomized controlled trials with longer follow-up and more diverse populations to establish whether the dietary and metabolic changes observed here can be produced and sustained.

## 5. Conclusions

In adults with obesity, aligning dietary guidance with energy-balance phenotypes was associated with significant reductions in total daily energy, carbohydrate and fat intake, and a greater relative contribution of protein. These macronutrient shifts occurred alongside phenotype-based behavioral guidance with no predetermined macronutrient targets. Due to the non-randomized, sequential design, these results reflect associations rather than demonstrated effects of phenotype tailoring itself. These findings support a personalized dietary approach in obesity management and warrant validation in larger, longer randomized controlled trials.

## Figures and Tables

**Figure 1:**
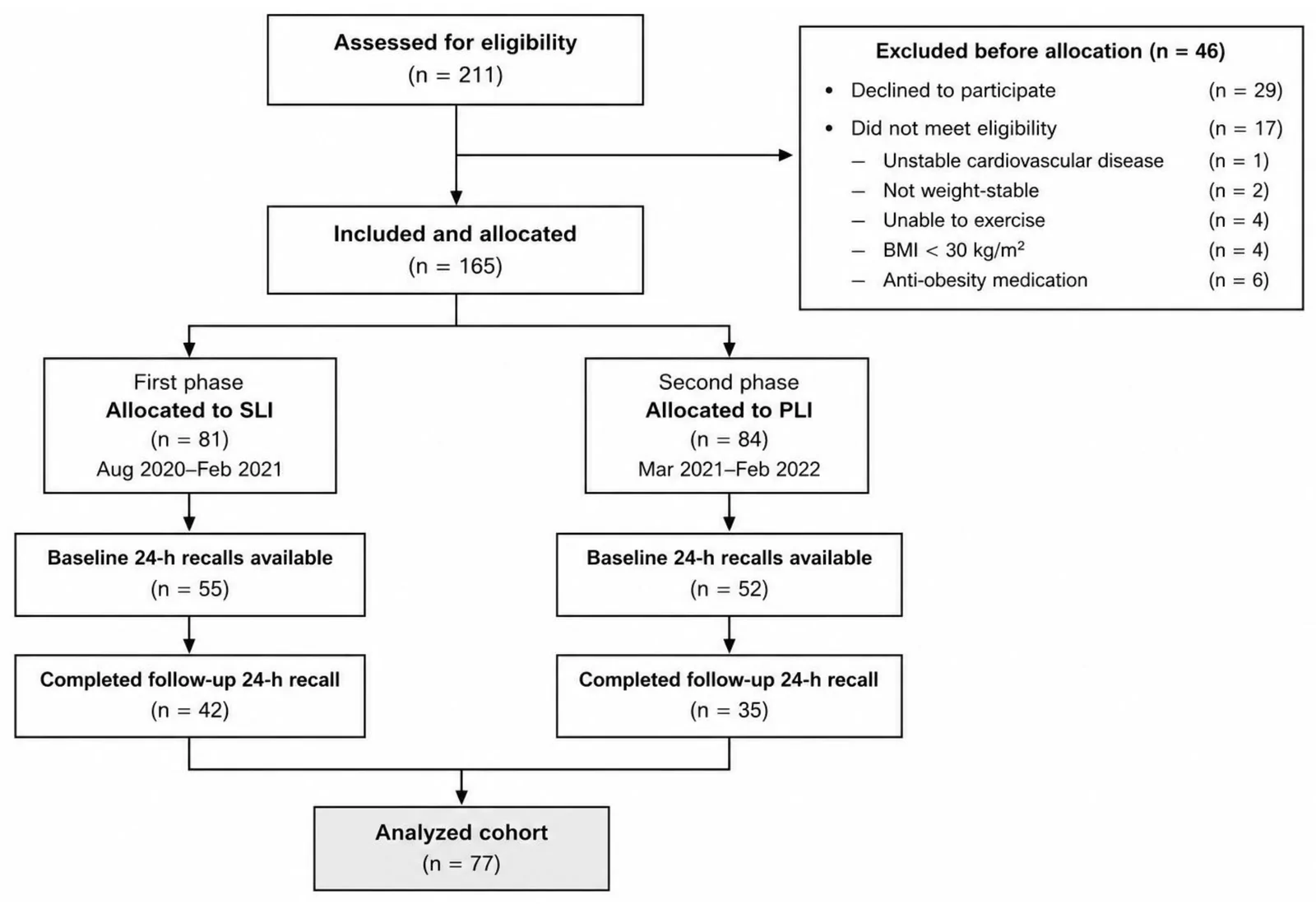
Participant flow diagram for the secondary analysis of the PHENO-Diet Trial. Eligible participants were adults aged 18–65 years with a BMI ≥ 30 kg/m^2^. Exclusion criteria were a BMI < 30 kg/m^2^, weight instability (>3% change in the preceding 3 months), unstable cardiovascular disease, inability to engage in physical activity, use of anti-obesity or other weight-affecting medication, and prior bariatric or endoscopic procedure. Counts for each exclusion reason are shown in the figure. PLI, phenotype-tailored lifestyle intervention; SLI, standard lifestyle intervention.

**Figure 2:**
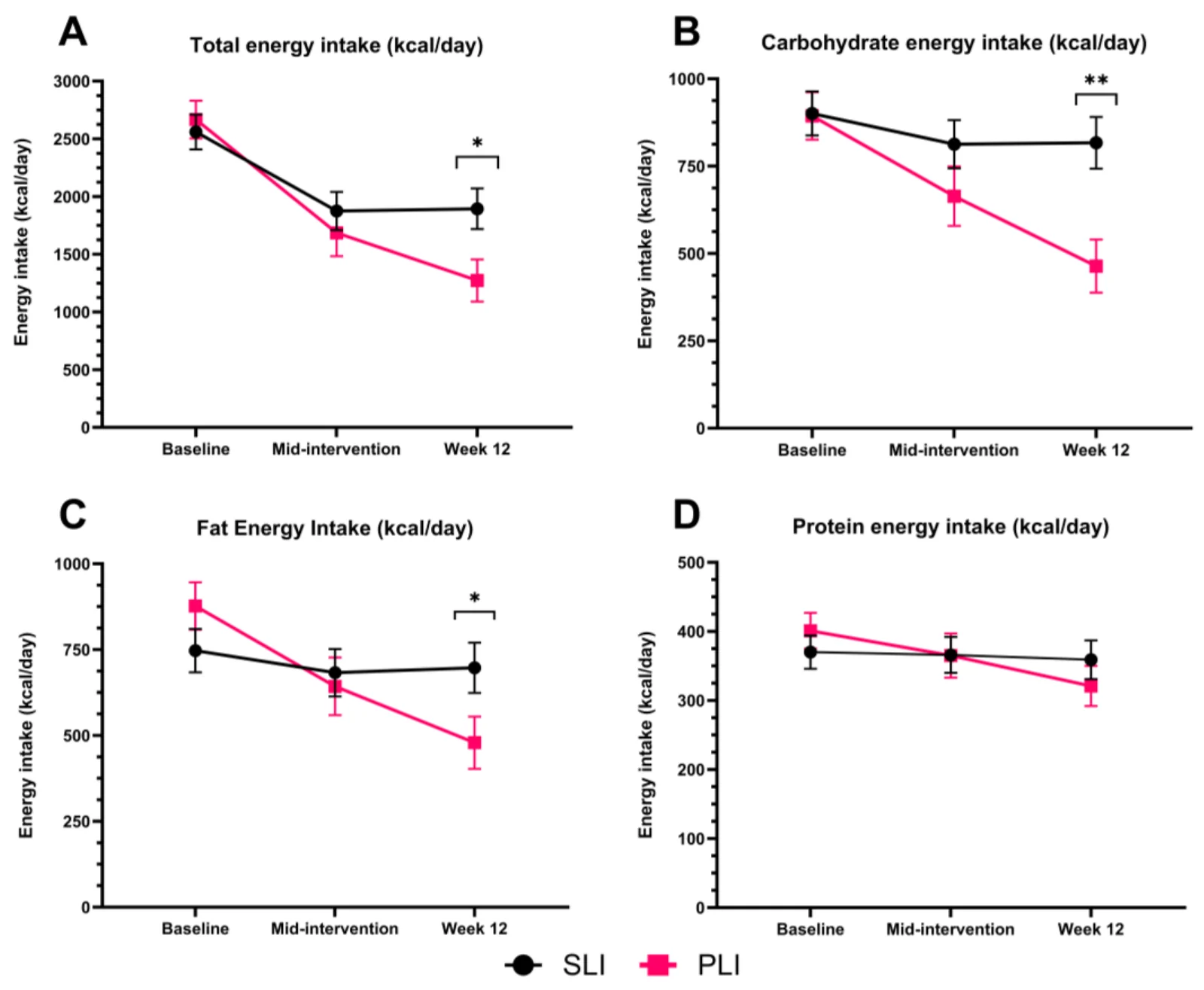
Changes in macronutrient intake (kcal/day). LSM ± SE of (**A**) total energy intake (kcal/day) and (**B**) carbohydrate, (**C**) fat, and (**D**) protein intakes (kcal/day) at baseline, mid-intervention and Week 12 in the SLI (black) and PLI (red) groups. Models were adjusted for age, sex and baseline weight as covariates. Asterisks denote FDR-adjusted (Benjamini–Hochberg) between-group differences at Week 12: *p* < 0.05 (*), *p* < 0.01 (**).

**Figure 3:**
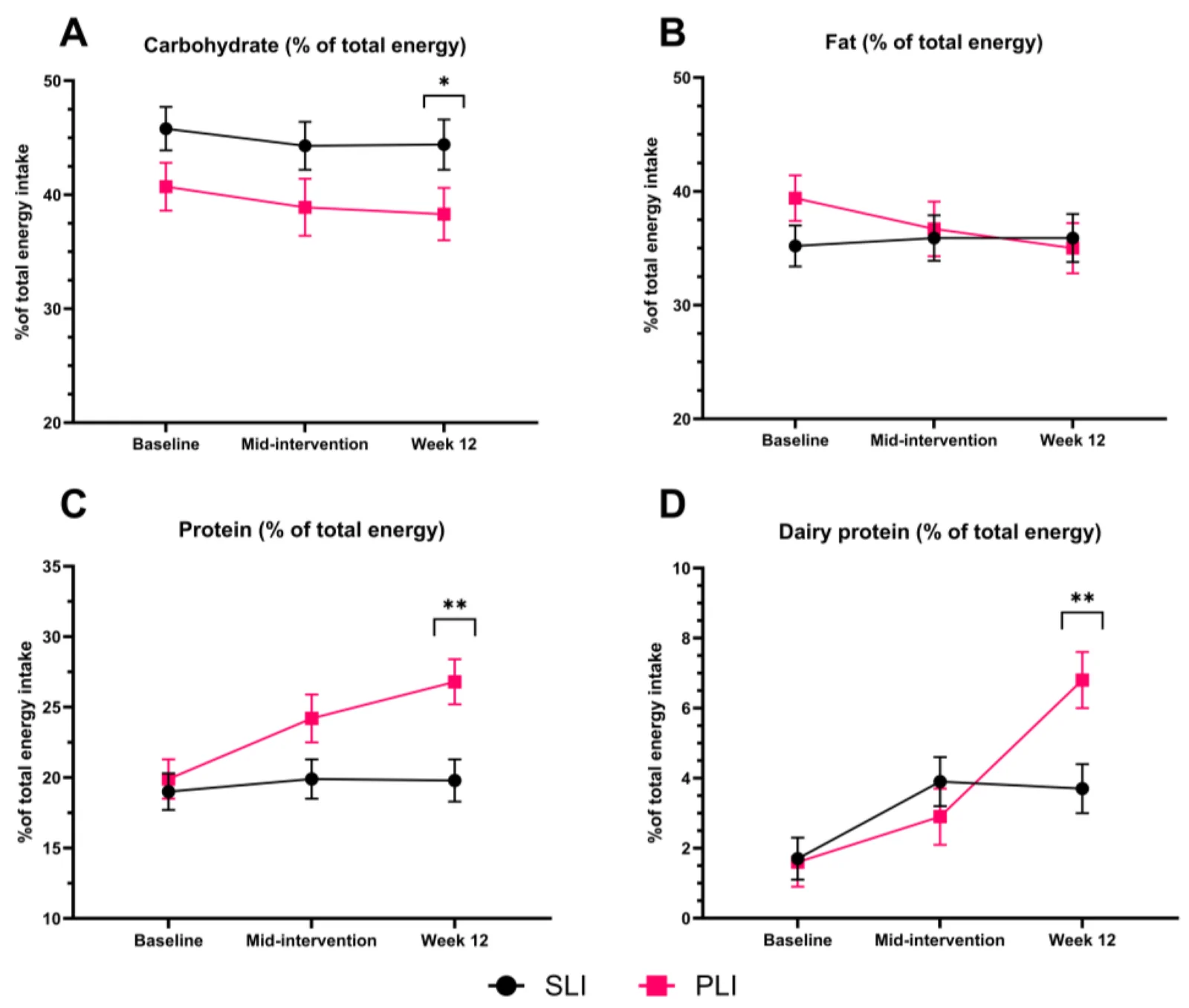
Changes in relative macronutrient composition (% of total energy). LSM ± SE of (**A**) carbohydrate, (**B**) fat, (**C**) protein and (**D**) dairy protein as % of total energy intake between SLI and PLI groups across timepoints. Model was adjusted for age, sex and baseline weight as covariates. Asterisks denote FDR-adjusted (Benjamini–Hochberg) between-group differences at Week 12: *p* < 0.05 (*), *p* < 0.01 (**).

**Figure 4:**
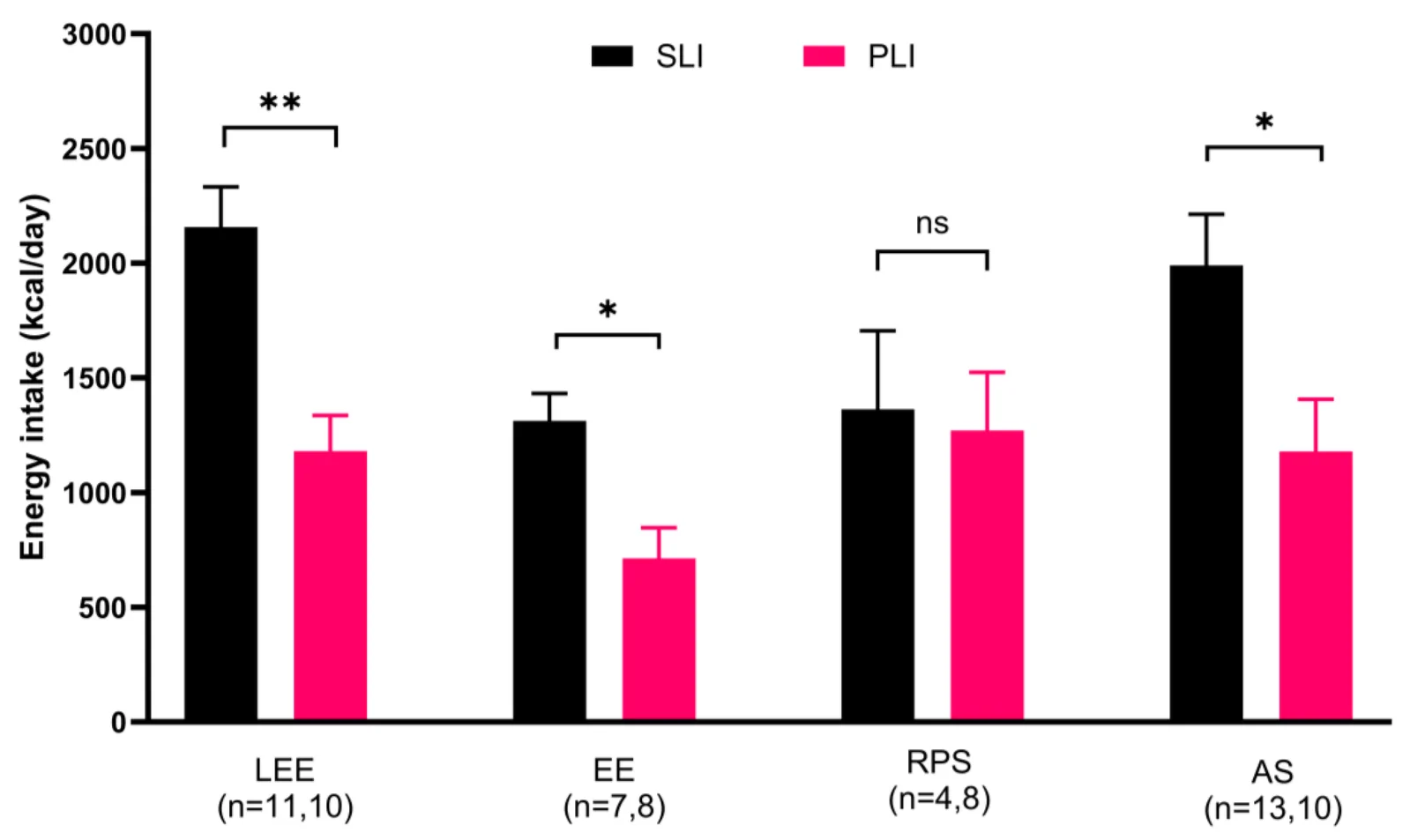
Total energy intake at week 12 by intervention group and phenotype. Total energy intake at week 12 adjusted for age, sex, baseline weight and baseline intake. LEE, low energy expenditure; EE, emotional eating; RPS, reduced postprandial satiety; AS, abnormal satiation; ns, not significant. Error bars represent standard error, and subgroup sizes are shown at the base of each bar. Asterisks denote between-group differences that remained statistically significant after Benjamini–Hochberg correction for multiple comparisons (* adjusted *p* < 0.05; ** <0.01). Corresponding numeric values appear in Table S5 of the Supplementary Material.

**Table 1:** Baseline Characteristics by Intervention Group.

Variable	SLI (n = 42)	PLI (n = 35)	*p*-Value	SMD (PLI-SLI)
Demographics				
Age (years) ^a^	43.9 (11.1)	45.2 (13.0)	0.66	0.11
Female sex, n (%) ^d^	35 (83.3%)	29 (82.9%)	1.00	−0.01
White race, n (%) ^d^	40 (95.2%)	32 (91.4%)	0.65	−0.15
Anthropometrics				
Weight (kg) ^b^	101.5 (95.9, 117.3)	101.0 (93.9, 124.0)	0.72	0.21
BMI (kg/m^2^) ^b^	36.2 (33.3, 42.4)	36.0 (33.5, 40.7)	0.97	0.02
Waist circumference (cm) ^b^	114.5 (107.7, 124.8)	111.3 (105.5, 121.7)	0.41	−0.04
Hip circumference (cm) ^b^	125.0 (116.0, 137.5)	123.0 (118.9, 135.1)	0.99	0.04
Total fat mass (kg) ^b^	48.6 (39.4, 60.2)	48.2 (42.1, 65.3)	0.66	0.16
Total lean mass (kg) ^b^	50.8 (46.1, 54.8)	52.5 (47.4, 60.1)	0.40	0.15
Metabolic markers				
Fasting glucose (mg/dL) ^b^	100.5 (95.5, 107.8)	97.0 (89.0, 106.0)	0.09	−0.43
HbA1c (%) ^a^	5.49 (0.47)	5.39 (0.52)	0.47	−0.20
Fasting insulin (µIU/mL) ^b^	8.9 (6.3, 15.4)	8.7 (5.2, 11.9)	0.31	−0.37
Cardiovascular Parameters				
Heart rate (bpm) ^a^	79.8 (11.1)	79.0 (9.6)	0.72	−0.08
Systolic BP (mmHg) ^b^	118 (112, 125)	118 (114, 127)	0.57	−0.03
Diastolic BP (mmHg) ^b^	80 (76, 86)	79 (74, 83)	0.44	−0.28
Lipids & Inflammation				
LDL cholesterol (mg/dL) ^a^	138.1 (40.7)	138.8 (36.7)	0.95	0.02
HDL cholesterol (mg/dL) ^b^	50 (41, 56)	47 (41, 57)	0.93	−0.14
Triglycerides (mg/dL) ^b^	108 (85, 158)	116 (87, 152)	0.95	−0.11
CRP, high sensitivity (mg/L) ^b^	3.4 (1.3, 5.8)	3.3 (2.5, 5.6)	0.75	0.09
Energy Balance & Behavior				
Energy intake to satiation (kcal) ^b^	830.2 (674.8, 1117.6)	855.9 (715.9, 1094.0)	0.79	0.04
Gastric emptying t½ (min) ^a^	134.4 (36.1)	121.4 (32.5)	0.10	−0.38
REE measured (kcal/day) ^a^	1721.2 (320.9)	1800.2 (333.3)	0.34	0.24
REE (% predicted) ^a^	95.2 (13.0)	97.8 (12.2)	0.43	0.21
HADS anxiety score ^b^	3.5 (1.8, 6.0)	6.0 (2.0, 8.0)	0.41	0.22
Phenotypes				
Abnormal satiation (%) ^c^	41.5	42.9	0.90	0.03
Reduced postprandial satiety (%) ^d^	11.9	28.6	0.09	0.42
Low energy expenditure (%) ^c^	35.7	44.1	0.46	0.17
Emotional eating (%) ^c^	21.4	31.4	0.32	0.23
Completers (%) ^d^	97.6	91.4	0.32	−0.27

^a^ *t*-test. ^b^ Wilcoxon rank-sum test. ^c^ Chi-square test. ^d^ Fisher’s exact test. Abbreviations: BMI (Body Mass Index), BP (blood pressure), CRP (C-reactive protein), REE (resting energy expenditure), SMD (standardized mean difference). Reference ranges: fasting glucose 70–99 mg/dL; HbA1c < 5.7%; fasting insulin 2.6–24.9 µIU/mL; LDL-C < 100 mg/dL (optimal); HDL-C ≥ 40 mg/dL (men)/ ≥ 50 mg/dL (women); triglycerides < 150 mg/dL.

**Table 2:** Baseline Total Calorie, Macronutrient and Dairy Intake.

Variable	SLI (n = 42)	PLI (n = 35)	*p*-Value
Energy intake (kcal/day) ^a^	2375.6 (1641.1, 3464.7)	1982.2 (1619.8, 3073.1)	0.363
Protein intake (kcal/day) ^a^	372.8 (288.8, 475.2)	296.6 (231.7, 419.7)	0.667
Fat intake (kcal/day) ^a^	731.7 (504.0, 1044.9)	596.3 (358.4, 849.4)	0.733
Carbohydrate intake (kcal/day) ^a^	790.4 (532.4, 1146.4)	709.8 (570.6, 1119.0)	0.297
Dairy protein intake (g/day) ^a^	0 (0, 14)	0 (0, 11)	0.981
Dairy protein (% of total energy) ^a^	0 (0, 2.52)	0 (0, 2.19)	0.991

^a^ Wilcoxon rank-sum test was used due to non-parametric distribution. Data expressed as median (IQR).

**Table 3:** Model-derived dietary intake by intervention group at baseline and week 12.

Outcome	SLI (LSM ± SE)		PLI (LSM ± SE)		Δ PLI − SLI (95% CI)	Unadjusted *p*-Value	FDR *p*-Value
	Baseline	Week 12	Baseline	Week 12			
Absolute intake (kcal/day)							
Total energy	2560 ± 151	1894 ± 176	2667 ± 164	1273 ± 182	−621 (−1086, −156)	0.009	0.018
Carbohydrate	901 ± 63	818 ± 73	894 ± 68	464 ± 76	−354 (−548, −160)	<0.001	0.002
Fat	747 ± 63	697 ± 73	877 ± 69	479 ± 76	−218 (−410, −26)	0.027	0.036
Protein	370 ± 24	359 ± 28	401 ± 26	321 ± 29	−38 (−110, +35)	0.306	0.306
Composition (% of total energy)							
Carbohydrate	45.8 ± 1.9	44.4 ± 2.2	40.7 ± 2.1	38.3 ± 2.3	−6.1 (−11.8, −0.4)	0.035	0.047
Fat	35.2 ± 1.8	35.9 ± 2.1	39.4 ± 2.0	35.0 ± 2.2	−0.9 (−6.4, +4.6)	0.760	0.760
Protein	19.0 ± 1.3	19.8 ± 1.5	19.9 ± 1.4	26.8 ± 1.6	+7.0 (+3.1, +10.9)	<0.001	0.003
Dairy protein	1.7 ± 0.6	3.7 ± 0.7	1.6 ± 0.7	6.8 ± 0.8	+3.1 (+1.1, +5.1)	0.002	0.004

Group values are LSM ± SE. Δ is the adjusted PLI − SLI difference at week 12 (95% CI); negative values indicate lower intake in PLI. *p*-values in the FDR column were adjusted (Benjamini–Hochberg) within each outcome family.

## Data Availability

Deidentified data underlying these results will be shared upon request from researchers with a methodologically sound proposal. Requests should be directed to acosta.andres@mayo.edu and will require a data access agreement.

## Supplementary Materials

The following supporting information can be downloaded at: https://www.mdpi.com/article/10.3390/nu18193159/s1, Table S1: Mixed model energy intake (kcal); Table S2: Mixed model macronutrient absolute intake (kcal); Table S3: Mixed model macronutrient intake proportion (% of total kcal); Table S4: Mixed model dairy protein; Table S5: Energy and macronutrient absolute intake (kcal) at 12 weeks by phenotype subgroup; Table S6: Baseline characteristics of participants included versus excluded from the dietary analysis; Table S7: Sensitivity analyses for the week-12 between-group difference in total energy intake; Table S8: Phenotype-specific PLI recommendations, physiological rationale, expected dietary shifts and observed outcomes.

## Abbreviations

The following abbreviations are used in this manuscript:

PLI	Phenotype-tailored lifestyle intervention
SLI	Standard lifestyle intervention
BMI	Body mass index
DXA	Dual-energy X-ray absorptiometry
LSM	Least squares mean
SE	Standard error
CI	Confidence interval
SMD	Standardized mean difference
FDR	False discovery rate
REE	Resting energy expenditure
