# Supplementary material for: Effect of a Phenotype-Tailored Lifestyle Intervention on Caloric Intake and Macronutrient Composition in Adults with Obesity: A Secondary Analysis of the PHENO-Diet Trial
Source: Nutrients. 2026 Sep 25;18(19):3159. doi: 10.3390/nu18193159 (PMC13666677; doi:10.3390/nu18193159)
Supplement: Supplementary file 1 [file nutrients-18-03159-s001.zip › nutrients-4518725-supplementary.pdf]

SUPPLEMENT

Table S1: Mixed Model Energy Intake (kcal)

Mixed-effects model adjusted for age, sex, and baseline weight; participant ID included as a random intercept.

Time-point	SLI LSM ± SE (kcal)	SLI (95% CI)	PLI LSM ± SE (kcal)	PLI (95% CI)	Δ (PLI – SLI) (95% CI)	Unadjusted p-value	FDR-adj p-value
**Baseline**	2560 ± 151	(2262, 2858)	2667 ± 164	(2343, 2991)	+107 (−294, +508)	0.599	
**Mid-intervention**	1875 ± 166	(1548, 2203)	1686 ± 203	(1285, 2086)	−190 (−670, +291)	0.436	
**Week 12**	1894 ± 176	(1547, 2241)	1273 ± 182	(914, 1632)	−621 (−1086, −156)	0.009	**0.018**
Values are least-squares means with 95% CI from a mixed-effects model adjusted for age, sex, and baseline weight (participant as random intercept). Δ is the PLI − SLI difference with 95% CI at each visit. Two p-values are shown. "Unadjusted p" is the raw between-group (PLI vs SLI) comparison at each visit. "FDR-adjusted p" is the Week-12 comparison after Benjamini–Hochberg correction for testing multiple outcomes							

Table S2: Mixed Model Macronutrient Absolute Intake (kcal)

Macronutrient	Time-point	SLI LSM ± SE (kcal)	SLI (95% CI)	PLI LSM ± SE (kcal)	PLI (95% CI)	Δ (PLI – SLI) (95% CI)	Unadjusted p-value	FDR p-value
**Carbohydrate**	Baseline	901 ± 63	(777, 1025)	894 ± 68	(758, 1029)	−7 (−175, +161)	0.930	
	Mid-intervention	813 ± 69	(676, 950)	664 ± 85	(497, 832)	−149 (−350, +53)	0.147	
	Week 12	818 ± 73	(672, 963)	464 ± 76	(314, 614)	−354 (−548, −160)	<0.001	**0.002**
**Protein**	Baseline	370 ± 24	(323, 418)	401 ± 26	(349, 453)	+31 (−33, +94)	0.339	
	Mid-intervention	366 ± 26	(314, 418)	365 ± 32	(303, 428)	−1 (−75, +74)	0.986	
	Week 12	359 ± 28	(304, 413)	321 ± 29	(265, 378)	−38 (−110, +35)	0.306	0.306
**Fat**	Baseline	747 ± 63	(622, 871)	877 ± 69	(742, 1013)	+131 (−36, +298)	0.124	
	Mid-intervention	683 ± 69	(547, 820)	643 ± 84	(476, 809)	−40 (−239, +159)	0.690	
	Week 12	697 ± 73	(552, 841)	479 ± 76	(329, 629)	−218 (−410, −26)	0.027	**0.036**
Values are least-squares means with 95% CI from a mixed-effects model adjusted for age, sex, and baseline weight (participant as random intercept). Δ is the PLI − SLI difference with 95% CI at each visit. Two p-values are shown. "Unadjusted p" is the raw between-group (PLI vs SLI) comparison at each visit. "FDR-adjusted p" is the Week-12 comparison after Benjamini–Hochberg correction for testing multiple outcomes.								

Table S3: Mixed Model Macronutrient Intake Proportion (% of total kcal)

Macronutrient	Time-point	SLI LSM ± SE (%)	SLI (95% CI)	PLI LSM ± SE (%)	PLI (95% CI)	Δ (PLI − SLI) (95% CI)	Unadjusted p-value	FDR p-value
**Carbohydrate**	Baseline	45.8 ± 1.9	(42.0, 49.6)	40.7 ± 2.1	(36.6, 44.8)	−5.1 (−10.1, −0.1)	0.044	
	Mid-intervention	44.3 ± 2.1	(40.3, 48.4)	38.9 ± 2.5	(34.0, 43.8)	−5.4 (−11.2, +0.4)	0.068	
	Week 12	44.4 ± 2.2	(40.1, 48.7)	38.3 ± 2.3	(33.8, 42.8)	−6.1 (−11.8, −0.4)	0.035	**0.047**
**Protein**	Baseline	19.0 ± 1.3	(16.5, 21.6)	19.9 ± 1.4	(17.2, 22.7)	+0.9 (−2.5, +4.4)	0.603	
	Mid-intervention	19.9 ± 1.4	(17.0, 22.7)	24.2 ± 1.7	(20.7, 27.6)	+4.3 (+0.2, +8.5)	0.041	
	Week 12	19.8 ± 1.5	(16.8, 22.8)	26.8 ± 1.6	(23.7, 29.9)	+7.0 (+3.1, +10.9)	<0.001	**0.003**
**Fat**	Baseline	35.2 ± 1.8	(31.6, 38.7)	39.4 ± 2.0	(35.5, 43.3)	+4.2 (−0.6, +9.0)	0.084	
	Mid-intervention	35.9 ± 2.0	(32.0, 39.8)	36.7 ± 2.4	(32.0, 41.5)	+0.9 (−4.8, +6.5)	0.767	
	Week 12	35.9 ± 2.1	(31.7, 40.0)	35.0 ± 2.2	(30.7, 39.3)	−0.9 (−6.4, +4.6)	0.760	0.760

Values are least-squares means with 95% CI from a mixed-effects model adjusted for age, sex, and baseline weight (participant as random intercept). Δ is the PLI − SLI difference with 95% CI at each visit. Two p-values are shown. "Unadjusted p" is the raw between-group (PLI vs SLI) comparison at each visit. "FDR-adjusted p" is the same Week-12 comparison after Benjamini–Hochberg correction for testing multiple outcomes.

Table S4: Mixed Model Dairy Protein

Time-point	SLI LSM ± SE (%)	SLI (95% CI)	PLI LSM ± SE (%)	PLI (95% CI)	Δ (PLI − SLI) (95% CI)	Unadjusted p-value	FDR p-value
Baseline	1.7 ± 0.6	(0.5, 3.0)	1.6 ± 0.7	(0.2, 2.9)	−0.2 (−1.8, +1.5)	0.860	
Mid-intervention	3.9 ± 0.7	(2.6, 5.3)	2.9 ± 0.8	(1.2, 4.6)	−1.0 (−3.0, +1.0)	0.305	
Week 12	3.7 ± 0.7	(2.2, 5.1)	6.8 ± 0.8	(5.2, 8.3)	+3.1 (+1.1, +5.1)	0.002	**0.004**

Values are least-squares means with 95% CI from a mixed-effects model adjusted for age, sex, and baseline weight (participant as random intercept). Δ is the PLI − SLI difference with 95% CI at each visit. Two p-values are shown. "Unadjusted p" is the raw between-group (PLI vs SLI) comparison at each visit. "FDR-adjusted p" is the same Week-12 comparison after Benjamini–Hochberg correction for testing multiple outcomes.

Table S5: Energy and macronutrient absolute intake (kcal) at 12 weeks by phenotype subgroup

Phenotype	Variable	SLI (LSM ± SE)	PLI (LSM ± SE)	β (95 % CI)	Unadjusted p-value	FDR p-value
**Low Energy Expenditure**	Total energy (kcal/day)	2157 ± 176	1181 ± 155	−976 (−1418 to −534)	<0.001	0.002
SLI = 11	Protein (kcal/day)	444 ± 40	274 ± 37	−170 (−270 to −70)	0.003	0.012
PLI = 10	Fat (kcal/day)	840 ± 113	469 ± 105	−372 (−654 to −90)	0.014	0.029
	Carbohydrates (kcal/day)	927 ± 82	482 ± 71	−445 (−666 to −222)	< 0.001	0.002
**Emotional Eating**	Total energy (kcal/day)	1312 ± 120	714 ± 133	−598 (−910 to −286)	0.004	0.014
SLI = 7	Protein (kcal/day)	265 ± 36	221 ± 38	−44 (−134 to +46)	0.385	0.513

PLI = 8	Fat (kcal/day)	477 ± 38	193 ± 40	−284 (−468 to −98)	<0.001	0.002
	Carbohydrates (kcal/day)	533 ± 77	325 ± 91	−208 (−442 to +26)	0.082	0.119
**Reduced Postprandial Satiety**<br>SLI = 4<br>PLI = 8	Total energy (kcal/day)	1363 ± 342	1271 ± 254	−91 (−933 to +750)	0.840	0.839
	Carbohydrates (kcal/day)	606 ± 139	494 ± 104	−112 (−456 to +232)	0.548	0.643
	Protein (kcal/day)	305 ± 88	374 ± 68	+69 (−152 to +289)	0.563	0.643
	Fat (kcal/day)	453 ± 122	395 ± 90	−58 (−356 to +240)	0.714	0.762
**Abnormal Satiation**<br>SLI = 13<br>PLI = 10	Total energy (kcal/day)	1990 ± 223	1179 ± 228	−810 (−1330 to −291)	0.007	0.019
	Carbohydrates (kcal/day)	783 ± 105	431 ± 104	−351 (−595 to −108)	0.012	0.027
	Protein (kcal/day)	339 ± 41	243 ± 44	−96 (−187 to −6)	0.053	0.085
	Fat (kcal/day)	875 ± 119	487 ± 128	−389 (−676 to −101)	0.017	0.030
**β (95 % CI)** represents the covariate-adjusted PLI − SLI difference at Week 12 from phenotype-specific multiple linear regressions (predictors: group, baseline intake, age, sex, baseline weight)<br>Covariate-adjusted least-squares means ± SE from phenotype-specific regressions (group, baseline intake, age, sex, baseline weight). β is the PLI − SLI difference at Week 12 with 95% CI; negative values indicate lower intake in PLI. "Unadjusted p" is the raw subgroup comparison; "FDR-adjusted p" applies the Benjamini–Hochberg correction across all 16 subgroup-by-outcome comparisons. Subgroup assignments are non-exclusive. Patients can have more than one phenotype.						

Table S6. Baseline characteristics of participants included versus excluded from the dietary analysis.

Variable	Included (n = 77)	Excluded (n = 88)	Test	p	SMD
**Demographics**					
**Age (years)**	44.5 ± 11.9	43.3 ± 12.0	t	0.52	0.10
**Female sex, n (%)**	64 (83.1)	70 (79.5)	Fisher	0.69	−0.09
**White race, n (%)**	72 (93.5)	79 (89.8)	Fisher	0.42	0.14
**Anthropometrics**					
**Weight (kg)**	109.4 ± 21.8	111.7 ± 22.1	Wilcoxon	0.28	−0.11
**BMI (kg/m²)**	37.8 ± 6.4	38.8 ± 6.6	Wilcoxon	0.20	−0.16
**Waist circumference (cm)**	115.8 ± 13.5	116.7 ± 13.9	Wilcoxon	0.60	−0.07
**Hip circumference (cm)**	127.8 ± 14.5	127.1 ± 13.9	Wilcoxon	0.66	0.05
**Total fat mass (kg)**	51.6 ± 14.2	52.9 ± 15.7	Wilcoxon	0.63	−0.09
**Total lean mass (kg)**	53.3 ± 9.3	55.2 ± 9.6	Wilcoxon	0.13	−0.20
**Metabolic markers**					
**Fasting glucose (mg/dL)**	100.1 ± 9.9	105.2 ± 21.8	Wilcoxon	0.57	−0.30

**HbA1c (%)**	5.4 ± 0.5	5.6 ± 0.7	t	0.09	−0.32
**Fasting insulin (µIU/mL)**	10.2 ± 6.6	12.0 ± 7.3	Wilcoxon	0.15	−0.25
**Cardiovascular**					
**Heart rate (bpm)**	79.4 ± 10.4	78.0 ± 11.1	t	0.38	0.14
**Systolic BP (mmHg)**	120.2 ± 10.8	121.2 ± 13.3	Wilcoxon	0.59	−0.08
**Diastolic BP (mmHg)**	80.5 ± 8.0	79.4 ± 9.2	Wilcoxon	0.56	0.13
**Lipids & inflammation**					
**LDL cholesterol (mg/dL)**	138.5 ± 37.8	136.8 ± 29.2	t	0.79	0.05
**HDL cholesterol (mg/dL)**	50.5 ± 13.7	50.8 ± 14.3	Wilcoxon	0.89	−0.02
**Triglycerides (mg/dL)**	121.2 ± 58.7	121.5 ± 53.0	Wilcoxon	0.82	0.00
**CRP (mg/L)**	4.3 ± 3.4	5.4 ± 4.9	Wilcoxon	0.20	−0.26
**Energy balance & behavior**					
**Energy to satiation (kcal)**	924.2 ± 364.9	862.7 ± 338.2	Wilcoxon	0.28	0.18
**Gastric emptying t½ (min)**	128.5 ± 34.9	130.8 ± 32.9	t	0.67	−0.07
**REE measured (kcal/day)**	1874.7 ± 361.7	1874.0 ± 305.8	t	0.99	0.00
**REE (% predicted)**	100.7 ± 14.1	98.9 ± 14.1	t	0.40	0.13
**HADS anxiety**	4.4 ± 3.2	4.5 ± 3.5	Wilcoxon	0.97	−0.03
**Phenotypes**					
**Abnormal satiation, n (%)**	32 (42.1)	26 (29.5)	chi-sq	0.09	0.26
**Reduced postprandial satiety, n (%)**	15 (19.5)	9 (10.2)	Fisher	0.12	0.26
**Low energy expenditure, n (%)**	30 (39.5)	38 (43.2)	chi-sq	0.63	−0.08
**Emotional eating, n (%)**	20 (26.0)	25 (28.4)	chi-sq	0.73	−0.05
**Intervention assignment**					
**PLI arm, n (%)**	35 (45.5)	49 (55.7)	Fisher	0.21	−0.21

Continuous variables summarized as mean ± SD; categorical variables as n (%). Between-group comparisons by t-test, Wilcoxon rank-sum, chi-square, or Fisher's exact test as appropriate. SMD = standardized mean difference (included − excluded). Included participants (n = 77) contributed a baseline and ≥ 1 follow-up 24-hour recall; excluded participants (n = 88) did not. No between-group difference was statistically significant

Table S7. Sensitivity analyses for week-12 between-group difference in total energy intake.

**Analysis**	**n**	**PLI − SLI difference, kcal/day (95% CI)**	**p-value**
Complete-case ANCOVA (week-12 completers)	56	−631 (−917, −346)	< 0.001

ANCOVA with jump-to-reference imputation	64	−487 (−755, −218)	< 0.001
All models adjusted for age, sex, and baseline weight (and baseline intake). The complete-case analysis was restricted to participants with a week-12 recall. The pattern-mixture (jump-to-reference) analysis imputed missing week-12 values in the PLI arm at the observed SLI mean.			

Table S8. Phenotype-specific recommendations, physiological rationale, and observed macronutrient outcomes in the phenotype-tailored lifestyle intervention (PLI)

Phenotype	PLI recommendation	Physiological rationale	Observed outcome (12 weeks)
Abnormal satiation	Low-calorie volumetric diet; high fiber, fruits and vegetables; 8-hour time-restricted eating; healthy second servings permitted	Reduce caloric intake window; maximize gastric distension to induce fullness; reduce energy density without restricting volume	−810 kcal/day vs. SLI (95% CI −1330 to −291); FDR p = 0.019; n = 10 PLI, 13 SLI
Reduced postprandial satiety	Low-calorie high-protein diet; pre-meal protein shake or snack; 3–5 structured meals/day	Protein preloads increase early GLP-1/PYY release and slow gastric emptying, targeting accelerated gastric emptying	No significant between-group energy difference (−91 kcal/day; p = 0.839); n = 8 PLI, 4 SLI
Low energy expenditure	Low-calorie high-protein diet; post-workout protein shake; resistance training + HIIT 4–5×/week; weekly physiotherapist check-in	Increase lean mass to boost REE; lean mass is the primary driver of resting energy expenditure	−976 kcal/day vs. SLI (95% CI −1418 to −534); FDR p = 0.002; largest reduction of any phenotype; n = 10 PLI, 11 SLI
Emotional eating	Balanced low-calorie diet; 3 structured meals/day; no snacks; 12-week CBT group telehealth sessions; mindfulness, emotion regulation, harm reduction	Avoid using food as a coping mechanism; address anxiety and reward-seeking eating behaviors through behavioral intervention	−598 kcal/day vs. SLI (95% CI −910 to −286); FDR p = 0.014; with improvements in HADS-A and TFEQ-EE scores; n = 8 PLI, 7 SLI
SLI (reference arm, all participants)	500 kcal/day deficit from measured REE; low-calorie diet guided by US Dietary Association recommendations; ≥150 min/week moderate PA; 10,000 steps/day; 12 individual wellness coaching sessions	Standard-of-care individualized caloric deficit; no phenotype-specific dietary modification	Reference comparator arm

Phenotype-specific recommendations were derived from the published trial [1] and delivered by a multidisciplinary team of obesity experts. CBT, cognitive-behavioral therapy; FDR, false discovery rate; HADS-A, Hospital Anxiety and Depression Scale – Anxiety subscale; HIIT, high-intensity interval training; PA, physical activity; PLI, phenotype-tailored lifestyle intervention; REE, resting energy expenditure; SLI, standard lifestyle intervention; TFEQ-EE, Three-Factor Eating Questionnaire – Emotional Eating subscale.

All energy intake outcomes are least-square mean differences from mixed-effects models adjusted for age, sex, baseline weight and baseline energy intake. FDR-adjusted p-values (Benjamini–Hochberg) reported for phenotype subgroup comparisons. Sample sizes reflect participants with ≥1 dietary recall in each phenotype subgroup. Participants meeting criteria for more than one phenotype received recommendations aligned with their dominant phenotype; additional phenotype-specific guidance was provided where applicable. Subgroup assignments are non-exclusive and phenotype overlap may influence the observed dietary outcomes.

References

1. Cifuentes L, Ghusn W, Feris F, Campos A, Sacoto D, De la Rosa A, et al. Phenotype tailored lifestyle intervention on weight loss and cardiometabolic risk factors in adults with obesity: a single-centre, non-randomised, proof-of-concept study. EClinicalMedicine. 2023;58:101923. https://doi.org/10.1016/j.eclinm.2023.101923
